# Hints and Improvements in the Practice of Medicine and Surgery

**Published:** 1799-07

**Authors:** 


### HINTS AND IMPROVEMENTS

### IN THE PRACTICE OF

### MEDICINE AND SURGERY.

### *On the Treatment of Biliary Calculi, Stone and Gravel.*

IN our laſt Number, p. 394, we gave a ſhort account of the ſucceſsful treatment of biliary calculi, by the internal uſe of vitriolic ether and ſpirit of turpentine. We alſo mentioned that M. DURANDE, a French phyſician of Dijon, had firſt preſcribed this powerful remedy. In juſtice to our countryman, C. WHITE, of York, we ſtate that he originally diſcovered this remedy, in conſequence of the experiments he made, by diſſolving biliary calculi in a mixture, conſiſting of alcohol and turpentine.

M. Durande, however, has the merit of having firſt employed vitriolic ether in preference to alcohol, with almoſt uniform ſucceſs, and of having furniſhed us with a more detailed account of the remedy.

There is ſcarcely a diſeaſe, with the cauſe and origin of which we are ſo imperfectly acquainted, as that of biliary and urinary concretions; and, as all the remedies hitherto employed have ariſen from hypothetical indications, we need not be ſurpriſed at the want of ſucceſs in the treatment of this painful and formidable diſorder. Although it be certain, that the formation of concrete matter in the kidneys requires a particular diſpoſition of the body, without which it will not take place, yet it is equally certain, that occaſional cauſes co-operate, ſuch as the uſe of hard and indigeſtible food, a ſedentary life, exceſs in wines containing much acid, and particularly the want of diluent watery drink. If a perſon is once afflicted with renal calculi, urinary concretions will ſoon follow, if the ſmalleſt particles of the former deſcend through the ureters into the bladder; and there increaſe in ſize by combining with the ſediment precipitated from the urine, unleſs they be ſpeedily waſhed away by that fluid. And as every individual may obſerve a greater or leſs quantity of this precipitate in his urine, it is evident that urinary concretions may ariſe without any prediſpoſition to biliary calculi, if foreign bodies get into the bladder, and remain there long enough to combine with the urinary precipitate.

The ætiology in theſe caſes being ſo obſcure, that the practitioner is frequently led, not unlike the lame man, by the blind, in the dark, Dr. WILLICH was induced to preſcribe M. Durande's compoſition, in a recent caſe of a patient, who, for ſix years, had been afflicted with ſpaſmodic ſtrictures of the urethra, and extreme difficulty of voiding urine, accompanied with excruciating pain in the region of the kidneys, and frequently extending to the lower vertebra, and the whole abdomen. The patient being of the advanced age of eighty, yet of a robuſt conſtitution, having led a ſea-faring life, and been accuſtomed to ſtrong food and the uſe of ſpirituous liquors, the pre-diſpoſing cauſes to gravel were manifeſt; while the character of the diſeaſe was eſtabliſhed by the diſcharge of gravelly urine, mingled with blood and mucus.

As he had paſſed no urine for ſeveral days, and could not be prevailed upon to apply the catheter, or at leaſt try the uſe of bougies, Dr. Willich preſcribed as follows: R/. *Æther. vitr.* drachm iij. *ſpir. terebinth. pur.* drachm drachm ij. M.—Of this mixture, after gently ſhaking it every time previous to its uſe, ten drops were at firſt directed to be taken every hour; theſe were gradually increaſed to twenty drops: next day the patient was directed to take twenty-five drops every two hours, in cold water ſweetened with honey; and, after continuing this medicine for three days, obſerving a proper diet and regimen, keeping the body open by the plentiful uſe of prunes, apples, &c. and fomenting the abdomen with an infuſion of chamomile flowers, with the addition of one ounce of laudanum to every quart of the infuſion, the patient was not only relieved from pain, but on the third night (after having taken 35 drops of laudanum to allay the ſpaſmodic tendency of the ſyſtem, and procure ſleep) he involuntarily diſcharged ſuch a quantity of urine as muſt, according to the account given by his nurſe, have amounted to ſeveral gallons. However this may be, it is indiſputable that the relief in this caſe was principally owing to the *gradual* action of the compound.

The efficacy of this remedy has lately been ſufficiently confirmed by the reſpectable authorities of Dr. Starck and Profeſſor Soemmering, of Mayence, who have obſerved its ſalutary effects in a variety of caſes, in which this compoſition, after it had been uſed for a few days only, relieved the moſt diſtreſſing ſymptoms, and cauſed the patients to diſcharge urinary calculi in ſmall pieces.

### *On the poiſonous effects of Mineral Acids*

In the '*Recueil Periodique*' No. xxxi. publiſhed by the Medical Society of Paris, we meet with an intereſting article, communicated by J. B. Desgranges, phyſician at Morges, in Switzerland. The author ſtates the caſe of a ſoldier, who, by miſtaking it for brandy, had ſwallowed a glaſs-ful of the ſulphuric acid; and, in conſequence of its violent action, was carried to the hoſpital, where the author found him in the agonies of death. He inſtantly preſcribed the following antidote, the good effects of which he had frequently witneſſed: viz. a drachm and a half of the carbonate of magneſia (magneſia uſta aërata) diſſolved in a teacup-ful of pure water. This doſe produced exceſſive vomiting; and, after repeating the magneſia in the quantity of half a drachm, every half hour, for about half a dozen times, and giving at intervals the ſolution of gum arabic with ſugar, the patient was completely recovered.

Deſgranges mentions, among other caſes, that of an artiſt, 36 years of age, who, in the moment of deſpair, ſwallowed more than half a wine-glaſs-ful of nitrous acid; he immediately felt a violent heat and irritation, extending from the throat to the ſtomach. When the author arrived, (the time not being ſpecified) his patient was ſeized with convulſive vomiting, and other ſymptoms, ſimilar to thoſe of the ſoldier mentioned in the preceding caſe: he inſtantly preſcribed a drachm of pure magneſia, with ſome ſugar diſſolved in water, aud ſoon after it half a drachm, which ſtopped the vomiting. He then directed a ſcruple to be taken every half hour, which, in leſs than three hours, relieved the patient from pain: on the following day, there was a conſiderable ſwelling and tenſion in the inner part of the throat, difficulty of breathing, painful deglutition, and gangrenous ſpots appeared about the mouth. The author, on this occaſion, obſerves, that this was a real gangrenous quinſey, but produced by a cauſe merely accidental, or factitious. He conſequently bled the patient twice within leſs than twelve hours, one of which bleedings was performed on the foot. At length, after the application of of mild purgative clyfters, and diaphoretics, to abate the miliary eruption on the fkin; which was accompanied with intolerable itching, and which took place about the fixth day after the accident, the patient was effectually cured.

The author's fifth obfervation relates to a fingular cafe of a young woman, who had been imprudent enough to rub her hair with a pomatum, of which arfenic formed an ingredient, with a view to deftroy vermin. Six days after ufing this pomatum, the whole head of the patient became fwelled, and fhe fuffered the moft excruciating pains over the whole body. Defgranges was confulted; he bled her freely, efpecially on the foot; directed chicken-broth for her drink, with a fmall portion of nitre; frequent wafhings with a decoction of lintfeed; mercurial honey; cordials; warm bathing of the lower extremities, &c. &c. Befides thefe remedies he caufed her head to be rubbed with *Baumé's pomatum*, made of cream and a fourth part of the powder of white chalk. He next applied from eight to ten leeches to the thighs, which produced an uneafy night; the fwelling of the head appeared to have increafed; and in the morning the whole body was covered with a confiderable eruption of fmall fpots, with white points refembling millet, extending even to the hands and feet. In this extremity the author again had recourfe to his favourite remedy, and ordered his patient repeated dofes of calcined magnefia, with the fyrup of marfh mallows, "which obvioufly increafed the evacuations of the primæ viæ." In fhort, the eruption fubfided, and all the alarming fymptoms abated in forty-eight hours, fo that the patient was declared out of danger in eight days.

M. Defgranges accompanies thefe cafes with fome defultory remarks on the imminent danger attending the external application of arfenic; and he obferves one of its conftant effects is, that the bodies of thofe who ufe it, are uniformly covered with blotches.—As to his liberal adminiftration of the magnefia, we have no other objection than what has been already pointed out by the editor of the 'Recueil Periodique,' that magnefia may not always be convenient; and that, delay in thefe cafes being attended with danger, there is a remedy more fimple and equally effectual, one which will be found in or near every cottage as well as in the palaces of the great—that is, *fimple water*, taken in *fufficient quantity*, which will not only dilute the moft virulent poifon, and reduce its acrimony fo far as to blunt the fharp points, for inftance, of arfenic,—but it will likewife inundate the ftomach as well as the inteftines, and operate either upwards, as an emetic, or as a cathartic, carrying the deleterious matter through the inteftinal canal, without much injury to the coats of the ftomach or bowels.

### *On the Treatment of Gangrene and Sphacelus.*

In a late treatife, publifhed by C. White, of York, containing remarks on fphacelus, particularly that fpecies which is accompanied with convulfive fymptoms, and has arifen from local external injury, we meet with a new method of cure propofed for that complaint. It confifts of a combination of mufk with fal cornu cervi volatilis, in equal proportions. The patient begins by taking 10 grains of each, or 20 grains of the compound, and increafes this dofe gradually to 80 or 100 grains, and upwards, of each. The effects of this medicine are ftated to be fuch as deferve the greateft attention of practitioners; but we do not find that it has been generally employed, unlefs by foreign practitioners, who fpeak of it in the higheft terms of commendation.

Another remedy of uncommon efficacy in gangrene and malignant inveterate ulcers, ftands recorded on the authority of Dr. Collin, of Vienna, who affirms that he found it equally fuccefsful in both, humid and dry fpecies of of ſphacelus. In the former, he directed the ſphacelated part to be covered with the powder of camphor, pretty thick; and in the latter caſe, to apply a ſtrongly camphorated liniment. See *Geſenius Handbuch der practiſchen Arzneymittellehre.* Edit. 2. p. 328.

The ſuperior efficacy of this remedy is further atteſted by the celebrated veteran, C. C. Hoffman, of Mayence, who has frequently preſcribed it with ſingular ſucceſs in the confluent ſmall-pox, when little hope remained of the recovery of the patient; and in the caſe of a German lady of high rank, after the cadaverous fetor had driven all her relations from the palace, he within twelve days uſed no leſs than *eighty ounces of camphor,* partly by giving a ſcruple every half hour internally, and partly by keeping the patient continually covered with camphorated liniment. C. Monch, another German phyſician, in a ſimilar manner, employed not leſs than *ten pounds and five ounces* of camphor, within twenty-two days, in the caſe of a young woman, twenty-two years of age, labouring under a confluent though benign ſpecies of ſmall-pox, without giving ſuch large doſes internally as in the preceding caſe treated by Dr. Hoffmann; but Monch's patient died, notwithſtanding that prodigious quantity of camphor had been uſed, and the whole body was covered with a dark-brown cruſt. *Ibid.* p. 326.

---

In our ſecond Number we gave a ſhort account of various new methods deviſed for treating inveterate ulcers, in which we particularly mentioned (p. 188.) the excellent antiſeptic qualities of the *Anguſtura bark.* Dr. Willich has ſince preſcribed it, in two caſes, with obvious good effect. One of his patients was a lady, in the primary ſtage of phthiſis pulmonalis, who had been troubled, for ſeveral months, with an ulcer on each of the heels, immediately below the ancle, the conſequence of miſmanaged chilblains, both being extremely painful, of a livid hue, and ſphacelous appearance: the other patient was a ſeafaring man, of a ſcorbutic taint, about fifty years of age, who, from an external injury which he had received many years ago, was afflicted with ſeveral inveterate and troubleſome ulcers, extending from the ancle to the inner part of the calf, on the right leg.—By a ſtrict attention to *diet* and *regimen,* and the external application of the Anguſtura bark, in powder, by covering the ulcers completely, and renewing the dreſſings twice a day, the former patient was relieved in leſs than three weeks, while the ulcers of the latter did not ſhow a diſpoſition to heal and to cicatrize till he had uſed the powder for upwards of a month, ſo that in about ſix weeks they were completely healed.

Although two caſes of this nature, imperfectly related, do not decide the value of a medicine, which is already, and more forcibly, recommended by great authorities; yet, Dr. Willich is of opinion, that the Anguſtura bark certainly deſerves to be more frequently employed than it is at preſent; for it appears to be almoſt entirely neglected or forgotten. Perhaps this may be aſcribed to the circumſtance, that it has, like moſt other powerful remedies, within theſe few years been converted into a kind of noſtrum—a quack-medicine—which could not fail to be frequently miſuſed, or miſapplied, by the ignorant multitude.

